# GDF15 – A potential novel biomarker of cognitive impairment in cervical dystonia

**DOI:** 10.3389/fnins.2026.1771471

**Published:** 2026-03-12

**Authors:** Artur Drużdż, Małgorzata Dudzic, Joanna Poszwa, Anna Rajewska, Aleksandra Mikołajczak, Wojciech Kozubski, Jolanta Dorszewska

**Affiliations:** 1 Department of Neurology, Municipal Hospital in Poznan, Poznan, Poland; 2 Laboratory of Neurobiology, Department of Neurology, Poznan University of Medical Sciences, Poznan, Poland; 3 Department of Laboratory Diagnostics, Municipal Hospital in Poznan, Poznan, Poland; 4 Department of Neurology, Poznan University of Medical Sciences, Poznan, Poland

**Keywords:** biomarker, botulinum toxin, cervical dystonia, cognitive decline, GDF15

## Abstract

**Introduction:**

Cervical dystonia (CD) is a chronic movement disorder characterized by motor symptoms and a spectrum of non-motor features, including cognitive difficulties, which may affect quality of life. To date, no biomarker exists to identify or monitor cognitive impairment in CD. Growth differentiation factor 15 (GDF15) has been associated with cognitive decline in several neurodegenerative diseases and movement disorders, providing a rationale for its investigation in CD. This study aimed to evaluate whether plasma GDF15 concentrations are associated with cognitive performance in individuals with CD and to assess examine the potential of GDF15 as a biomarker of cognitive decline in CD.

**Methods:**

Plasma GDF15 levels were measured in patients with CD before and after botulinum toxin (BoNT) treatment and compared with healthy controls. Correlations between GDF15 concentration, cognitive performance (MoCA total and domain scores), and clinical characteristics—including age, disease duration, and TWSTRS motor severity—were analyzed using Spearman's and Pearson's coefficients.

**Results:**

GDF15 concentrations were significantly higher in individuals with CD compared with healthy controls. However, GDF15 levels showed no association with age, disease duration, symptom severity, or treatment response, and remained stable following botulinum toxin administration. No correlations were found between GDF15 and global cognitive performance, with the exception of a correlation with the visuospatial MoCA sub score.

**Discussion:**

While GDF15 concentrations were elevated in CD, they did not demonstrate consistent relationships with clinical features or cognitive outcomes. These findings suggest that GDF15 cannot currently be considered a reliable biomarker of cognitive impairment in CD. Given the limited sample size, this study should be regarded as preliminary.

## 1 Introduction

Cervical dystonia (CD) is a chronic neurological movement disorder characterized by sustained or intermittent involuntary contractions of the cervical musculature, resulting in abnormal head postures and often associated pain (Albanese et al., 2023). Although motor symptom severity represents the core clinical feature of CD, an increasing body of evidence indicates that non-motor manifestations – such as anxiety, depression, sleep disturbances, and cognitive deficits – constitute independent components of the disorder (Peall et al., 2023). The impact of both motor and non-motor symptoms on daily functioning has been well documented, and it is now increasingly recognized that they are critical determinants of health-related quality of life in individuals with CD (Junker et al., 2021). In view of the wide-ranging clinical manifestations of CD, the investigation of molecular markers that may contribute to its motor and non-motor characteristics has become increasingly relevant. Growth differentiation factor 15 (GDF15), a cytokine associated with mitochondrial stress, inflammation, and cellular injury, has been linked to cognitive impairment and disease burden in several neurodegenerative and movement disorders.

GDF-15 is a protein belonging to the transforming growth factor β (TGF-β) family that is linked to stress response. It is initially produced as a 35 kDa precursor (pro-GDF15) and after dimerization and proteolytic cleavage, it is secreted into the bloodstream as a mature 25-kDa homodimer (Li et al., 2024). It plays a role in essential biological processes, such as the proliferation of hematopoietic cells, the regulation of energy balance, and the metabolic activity of adipose tissue. As a stress cytokine, it plays a protective role by reducing excessive inflammatory signaling and maintaining tissue homeostasis. It primarily binds to the GRFAL receptor, which activates signaling pathways including MAPK/ERK, PI3K/AKT, and JAK/STAT, involved in controlling various cellular processes such as proliferation, migration, differentiation, survival, and apoptosis (Guo et al., 2020).

GDF15 is naturally expressed at low levels in various cell types, including neurons, glial cells, as well as in the liver, lungs, pancreas, kidneys, heart, and tumor tissues. Its plasma levels increase during immune-mediated stress responses, not only in various diseases, including cancer, autoimmune diseases, cardiovascular diseases, obesity, and neurological conditions, but also due to physiological stressors such as aging, as well as in psychological stress (Sigvardsen et al., 2025).

Despite the fact that CD is not generally regarded as a neurodegenerative condition, its clinical spectrum encompasses non-motor manifestations, including cognitive complaints, which may involve stress-related biological pathways. Therefore, investigation of GDF15 concentrations in this population may help clarify whether such molecular mechanisms contribute to the non-motor dimensions of CD and to evaluate the potential relevance of GDF15 as a biomarker in this rare disorder.

The objective of this study was to determine whether GDF15 could serve as a potential biomarker for cognitive impairment in CD.

### 1.1 Clinical rationale for the study

Cognitive difficulties are now widely recognized as an additional non-motor feature of CD (Defazio et al., 2024). However, there is currently no biomarker that can be used to identify or monitor cognitive decline in this patient population. GDF15 has been proposed as a marker of cognitive impairment in several neurodegenerative diseases and movement disorders, supporting its investigation in CD. Evaluating GDF15 concentrations in this context may therefore help determine whether this molecule holds potential as an indicator of cognitive dysfunction in CD.

## 2 Materials and methods

### 2.1 Study design and bioethics

A prospective, controlled, observational, and clinical pilot study was performed. The Strengthening the Reporting of Observational Studies in Epidemiology (STROBE) cohort reporting guidelines were utilized in this study. The study was conducted in accordance with the principles established by the Declaration of Helsinki. Prior to the initiation of the study, it received approval from the Institutional Ethics Committee of the Poznań University of Medical Sciences (protocol code 525/23, approved on June 29, 2023). Participation in the study was voluntary. The study's design and purpose were explained to all participants, after which written consent was obtained from each individual.

### 2.2 Study participants

A total of 32 patients, ranging in age from 18 to 65 years, diagnosed with idiopathic cervical dystonia were enrolled in the study. These patients were part of the Polish National Health Care Treatment Program for focal dystonia, treated with botulinum toxin (BoNT), and had received stable BoNT doses for a minimum of 0.5 years prior to the study. Exclusion criteria encompassed hypersensitivity to BoNT or its components, prior deep brain stimulation implantation, and comorbidities that could potentially influence study outcomes. These comorbidities included clinically significant visual impairment, heart failure (New York Heart Association class II/III or higher), mobility impairment, chronic kidney disease (estimated glomerular filtration rate < 60 mL/min/1.73 m^2^), hepatic dysfunction, systemic inflammatory or malignant disease, alcohol dependence, impaired verbal communication, and psychiatric disorders that precluded neuropsychological assessment. A comprehensive medical history has been obtained from each participant. Particular emphasis was placed on excluding individuals with a diagnosis of schizophrenia or Alzheimer's disease, as well as individuals who have used antipsychotic medications, either currently or previously. The capacity to provide informed consent was obligatory.

A control group of 23 healthy, age- and sex-matched volunteers without dystonia was recruited using identical exclusion criteria to minimize potential confounding factors.

### 2.3 Clinical assessments

Participants diagnosed with dystonia were evaluated at two time points: on the day of the scheduled botulinum toxin (BoNT) injection, prior to treatment administration (baseline assessment), and 4–6 weeks following the injection (second assessment), corresponding to the period of maximum therapeutic efficacy commonly reported in clinical studies and trials. The control group underwent a single evaluation.

The study group was subjected to a detailed neurological examination conducted by a neurologist specialized in movement disorders and BoNT therapy. Both groups underwent cognitive function assessment and completed a standardized questionnaire designed to collect information on sociodemographic characteristics and medical history.

### 2.4 Cognitive assesment

The Montreal Cognitive Assessment (MoCA) has been utilized to evaluate cognitive functions. MoCA is a standardized tool used to screen for cognitive function in clinical and research settings. It evaluates multiple cognitive domains, including attention, executive function, memory, language, visuospatial skills, and orientation, using a 30-point scale. The assessment is administered in approximately 10 mins and has a cut-off score of 26, with scores below this indicating cognitive impairment. In this study, two alternative versions of the MoCA questionnaire (version 8.1 and 8.2) were utilized to minimize learning effects in the study group. These versions were employed to assess cognitive functions before and 4 to 6 weeks after botulinum toxin (BoNT) treatment. While learning effects are most significant with memory and executive function assessments, they are minimal or absent in mood and symptom self-reports, such as anxiety, sleep disturbances, and pain.

### 2.5 Analysis of GDF15 protein concentration

Biochemical analyses were performed to determine the concentration of GDF15 protein in patients with CD and control participants. Plasma GDF15 concentrations were measured using a commercially available immunoassay kit (ELISA)-Human GDF15 ELISA Kit (ELK2756 ELK Biotechnology CO., LTD, China)-according to the manufacturer's protocol (inter- and intra-assay variation coefficients were: < 10 % and < 8 %). The curve points, and each analyzed sample was assessed twice.

### 2.6 Statistical analysis

Statistical analyses were performed using Statistica software, version 13 (StatSoft, Kraków, Poland). Descriptive statistics were presented according to the type of variable: means with standard deviations (SD) for continuous variables, medians with ranges for ordinal variables, and counts with percentages for nominal variables. The Shapiro–Wilk test was used to assess the normality of data distribution.

Group comparisons were conducted using the Student's t-test for independent samples when data were normally distributed, and the Mann–Whitney U test when normality was not met or for ordinal data. The Pearson's chi-square test was applied for nominal variables. Within-group comparisons between the first and second measurements were performed using the paired Student's *t*-test for normally distributed data or the Wilcoxon signed-rank test otherwise. Spearman's rank correlation coefficient was used to assess associations between variables. A *p*-value of < 0.05 was considered statistically significant.

## 3 Results

### 3.1 Clinical characteristics

The socio demographic characteristics of the study and control groups are presented in Table 1.

**Table 1:** Sociodemographic characteristics of the studied population (study group and controls).

**Characteristics**	**Range**	**Study group (*n* = 32)**	**Control group (*n* = 23)**	***p*- value**
Age in years (Mean ± SD)	22-65	53.16 ± 11.00	49.96 ± 11.34	0.31
Education (n)	< 8yrs	2	0	0.70
	8-12 yrs	15	10	
	>12 yrs	15	13	
Gender n (%)	Female	26 (81)	20 (87)	0.71
	Male	6 (19)	3 (13)	
Disease duration (yrs ± [SD])	1-18	6.97 ± 4.73	-	-

### 3.2 GDF15 concentration in patients with cervical dystonia and controls

As shown in Table 2, patients with CD exhibited significantly higher mean serum GDF15 concentrations compared with healthy controls (1.81 ± 3.78 pg/ml vs. 0.63 ± 0.39 pg/ml; U = 495, *p* = 0.031). This difference was largely influenced by three patients with markedly elevated GDF15 values (>10 pg/ml). All three individuals fulfilled the study inclusion and exclusion criteria; none had a diagnosis of schizophrenia or Alzheimer's disease, none had a history of antipsychotic medication use, and none presented with clinically significant comorbidities known to affect GDF15 concentrations. Sensitivity analysis excluding these three cases demonstrated that the difference in mean GDF15 concentrations between groups remained statistically significant (0.61 ± 0.07 pg/ml vs. 0.53 ± 0.38 pg/ml; *p* = 0.026), although the magnitude of the difference was reduced by approximately 14%.

**Table 2:** GDF15 concentration in the study group: assessment 1. (pre-treatment), assessment 2. (at peak effect) and control group.

**Group**	**GDF15 concentration**					
	**N**	**Mean**	**Median**	**Minimum**	**Maximum**	**SD**
Study group: Assessment 1.	32	1.8122	0.6091	0.4470	14.4	3.7751
Study group: Assessment 2.	32	1.6076	0.6157	0.2703	15.8	3.5907
Control group	23	0.6343	0.5242	0.2392	2.139	0.3945

As demonstrated in Table 3, no statistically significant correlations were identified between GDF15 concentrations and clinical parameters (age, disease duration, disease severity) in any of the analyses conducted (all *p* >0.05). The correlation coefficients (ρ) ranged from – 0.086 to 0.905, indicating very weak associations between the variables. Age showed no correlation with GDF15 concentrations in either the dystonia group or the control group. Similarly, disease duration showed no association with GDF15 levels within the dystonia cohort. Symptom severity, as measured by the TWSTRS scale, was not found to correlate with GDF15 in either the first or the second assessment. Notably, the presence of outlier GDF15 values (>10) did not influence the outcomes. All findings were consistent across the two statistical approaches used: Spearman's rank correlation and Pearson's correlation.

**Table 3:** Results of Spearman's rank correlation analysis examining associations between GDF15 concentrations and clinical parameters in a study group (patients with dystonia) and in a control group.

**Variable 1**	**Variable 2**	**Rho (ρ)**	***p*-value**	**Direction**	**Strength**
**Study group** ***n*** = **32**					
Age	GDF15 assessment 1.	0.0716	0.6970	Positive	Very weak
Age	GDF15 assessment 2.	−0.0198	0.9141	Negative	Very weak
Disease duration	GDF15 assessment 1.	0.0083	0.9641	Positive	Very weak
Disease duration	GDF15 assessment 2.	0.0905	0.6222	Positive	Very weak
TWSTRS assessment 1.	GDF15 assessment 1.	−0.0032	0.9861	Negative	Very weak
TWSTRS assessment 2.	GDF15 assessment 2.	0.0668	0.7166	Positive	Very weak
**Control group** ***n*** = **23**					
Age	GDF15 assessment 1.	−0.0862	0.6959	Negative	Very weak

Age is expressed in years. Disease duration is presented in years. disease severity was assessed using the TWSTRS (Toronto Western Spasmodic Torticollis Rating Scale), with total scores ranging from 0 to 35 points. GDF15 concentrations are reported for the first (GDF15 assessment 1.) and second assessments GDF15 assessment 2. A p-value < 0.05 was considered statistically significant.

### 3.3 BoNT treatment effect

One of the primary research questions guiding this study was whether there is a difference in GDF15 concentration before and after toxin administration. As presented in Table 1, our results did not show significant difference in GDF15 concentrations in the study group at baseline and at the second assessment at peak effect time of botulinum toxin (*p* = 0.970).

### 3.4 GDF15 concentrations and MoCA score

The correlation between GDF15 plasma concentration and MoCA score (total score and specific cognitive domains scores) has been performed to verify whether circulating levels of GDF15 reflect the severity of cognitive impairment observed in patients with CD. The only correlation has been observed between plasma GDF15 concentration and the visuospatial subscore of the MoCA. No correlations were detected between GDF15 concentration and other MoCA subscales (Table 4).

**Table 4:** Correlations between GDF15 concentration and MoCA scores in the study group pre- and post- treatment and control group.

**GDF15 concentration and MoCA domain**	**Study group (*****n*** = **32) Pre-treatment**		**Study group (*****n*** = **32) Post-treatment**		**Control group (*****n*** = **23)**	
	**R**	**p**	**R**	**p**	**R**	**p**
**Total score**	0.050	0.784	0.145	0.428	−0.192	0.380
**Visuospatial**	**0.352**	**0.048** ^*^	0.174	0.340	−0.127	0.565
**Attention**	0.064	0.728	0.056	0.761	0.225	0.215
**Language**	0.300	0.095	−0.344	0.054	−0.289	0.181
**Abstract thinking**	0.045	0.805	0.235	0.195	−0.11	0.587
**Delayed recall**	−0.220	0.225	−0.220	0.225	0.009	0.969

## 4 Discussion

Although CD is primarily classified as a movement disorder, growing evidence indicates that non-motor symptoms—including cognitive dysfunction, depression, anxiety, and sleep disturbances—contribute substantially to the overall disease burden (Junker et al., 2021; Defazio et al., 2024; Han et al., 2020; Dudzic et al., 2025). GDF15 has recently emerged as a potential biomarker linked to neurodegeneration, inflammation, and stress-related neuronal processes, all of which may play a role in cognitive decline. Increasing research highlights an association between GDF15, a stress-induced cytokine, and cognitive deterioration observed in various neurodegenerative and movement disorders (Chen et al., 2024; Beydoun et al., 2023).

Elevated GDF15 levels in both blood and cerebrospinal fluid (CSF) have been repeatedly connected to the development and progression of dementia, characterized by a gradual decline in cognitive functions such as memory, attention, thinking, reasoning, and judgment (Xue et al., 2022). Neurodegenerative disorders are primarily characterized by the progressive loss of neurons, which in consequence lead to functional impairment of the nervous system. One of the major mechanisms contributing to this process is neuroinflammation, described as an inflammatory response within the central nervous system (CNS). It is particularly noteworthy that GDF15 is a protein that is involved in the modulation of inflammatory pathways, the dysregulation of which contributes to the pathogenesis of neurodegenerative diseases associated with dementia, including frontotemporal dementia (FTD) and Alzheimer's disease (AD). In patients diagnosed with FTD, higher baseline concentrations of GDF15 in peripheral blood have been found to be associated with faster cognitive decline in executive functions and more rapid progression of neurodegeneration (Chen et al., 2024). The most recent research indicate an existing gap in the field of molecular biomarkers of dementia from diverse origins (Poszwa et al., 2025). This observation points to GDF15 as a potential biomarker predicting adverse outcomes in FTD and suggests that GDF15-driven pathways may play a crucial role in the pathophysiology of disease progression. Conversely, emerging evidence from AD suggests that GDF15 may contribute to cognitive impairment by influencing mechanisms that regulate amyloid dynamics. Higher CSF levels of GDF15 have been observed in patients with lower Aβ 1-42, which may represent a compensatory response aimed at limiting Aβ aggregation and deposition, possibly through mechanisms involving microglial activation. Furthermore, in individuals diagnosed with AD, GDF15 levels in CSF have been found to negatively correlate with Mini-Mental State Examination (MMSE) scores, a widely used tool to examine cognitive performance. Mediation analysis revealed that the observed association between GDF15 levels and cognitive performance, measured by MMSE, is largely dependent on Aβ 1-42, implying that amyloid-related mechanisms may underlie this relationship in AD (Plantone et al., 2025). Interestingly, even among cognitively unimpaired individuals, higher circulating GDF15 levels have been associated with an increased risk of developing dementia in the future, as well as with smaller total brain and hippocampal volumes, greater white matter hyper intensity volume and poorer outcomes in global cognitive performance. Elevated GDF15 were also associated with poorer outcomes in tests of logical memory, executive function, and visuospatial processing (Plantone et al., 2025; McGrath et al., 2020; Guo et al., 2024).

Beyond dementia-related conditions, it should be emphasized that GDF15 also appears to be a promising marker of cognitive impairment in movement disorders. The primary research question guiding this study is whether GDF15 could serve as a potential biomarker for cognitive impairment in CD. The significantly higher GDF15 concentration identified in the study group indicate that dystonia, as a disorder, is associated with elevated GDF-15 levels, though there is considerable heterogeneity within the patient population. This is in line with the studies in other movement disorders, that consistently shows that serum concentrations of GDF15 are significantly elevated in patients with Parkinson's disease (PD) (Miyaue et al., 2020). It has been proposed that the elevated levels of serum GDF15 may reflect mitochondrial dysfunction in the skeletal muscle of PD patients, as secretion of GDF15 from muscle tissue has been detected in response to mitochondrial stress. However, the significant effect of age on GDF15 concentrations, which has been demonstrated to be positively correlated with advancing age, has not been confirmed in the present study (Miyaue et al., 2023). No correlation between age and GDF15 concentration has been identified in either the study cohort or the control group. In contrast, Maetzler et al. (2016) reported that higher CSF GDF15 concentrations were positively correlated with later age at disease onset and negatively correlated with MMSE scores, suggesting a potential relationship between this marker and both motor and cognitive aspects of Parkinson's disease (Maetzler et al., 2016).

Similar to PD, elevated serum GDF15 levels have been observed in atypical Parkinsonian syndromes, including progressive supranuclear palsy (PSP) and multiple system atrophy (MSA). GDF15 concentrations showed a positive correlation with age not only in PD but also in PSP and MSA (Miyaue et al., 2023). These findings suggest that, due to the similar levels of GDF15 observed across these conditions, the clinical differentiation between PD and atypical Parkinsonian syndromes based on this marker alone may be challenging. In both MSA subtypes - parkinsonian variant (MSA-P) and cerebellar variant (MSA-C) - the highest GDF15 levels were observed in male patients and in individuals with a disease duration exceeding 3 years. Previous studies have suggested that an older age of onset and the MSA-P subtype are associated with more severe motor and non-motor symptoms as well as poorer survival outcomes. However, when stratified by age of onset or clinical subtype, no significant differences in serum GDF15 levels were detected. The comparable GDF15 levels observed in both MSA subtypes are consistent with the shared underlying neurodegenerative pathology (Yue et al., 2020).

Studies assessing CSF GDF15 levels in diseases with underlying Lewy body pathology, have shown that concentrations vary with clinical severity The lowest values were observed in patients with early-stage PD without dementia (PDND), intermediate values in patients with dementia with Lewy bodies (DLB), and the highest values in patients with dementia in Parkinson's disease (PDD), who presented both severe motor and cognitive disorders. Notably, this evidence also indicated the potential of GDF15 to detect prodromal stages of disorders with underlying Lewy body pathology and to reflect the disease severity assessed by Hoehn and Yahr stages and MMSE - independently of disease duration (Maetzler et al., 2016).

Conversely, in the present study, the severity of the disease - dystonia symptoms measured in TWSTRS, has not been related to the GDF15 levels. The GDF15 concentration remained relatively stable over the observation period. Botulinum toxin—an effective symptomatic treatment targeting muscle overactivity—did not alter GDF15 concentrations, further suggesting that GDF15 is not directly linked to the motor expression or symptomatic modulation of CD. This would be consistent with the contemporary definition of dystonia, which is categorized as a movement disorder that originates from a neurocircuit dysregulation, as rather than a neurodegenerative process (Defazio et al., 2024; Yao et al., 2023).

GDF15 levels in patients with CD differed significantly from those observed in healthy controls, indicating that group-level differences may still be present even in the absence of relationships with clinical measures. In this study, GDF15 concentrations showed no associations with age, disease duration, symptom severity, or treatment response in CD, and remained stable between baseline and the peak effect of BoNT. Likewise, no correlations were found between GDF15 and overall cognitive performance, apart from a modest correlation with the visuospatial MoCA subscore. These findings do not align with the observations from other movement disorders and neurodegenerative diseases in which variability in GDF15 has been reported, suggesting that its role in CD may differ.

The observed association with the visuospatial MoCA subscore should be interpreted as exploratory and was no longer significant following effective BoNT treatment, supporting the view that cognitive alterations in cervical dystonia are subtle, non-progressive, and potentially modulated by secondary or state-dependent factors. This interpretation is consistent with accumulating evidence indicating that cognitive dysfunction in idiopathic adult-onset dystonia is generally mild, heterogeneous, and not indicative of a neurodegenerative process, with executive and visuospatial domains showing the most variable findings across studies (Defazio et al., 2024). Moreover, non-motor features such as psychological distress, mood and anxiety symptoms, social burden, and pharmacological treatment have been repeatedly shown to influence cognitive performance in dystonia and may act as important confounders (Bailey et al., 2022). Although these factors were not systematically assessed in the present study, they likely contribute to the modest and context-dependent cognitive findings observed and further support the exploratory nature of the reported associations.

A key limitation of this work is the relatively small sample size, which is partly due to the rarity of CD and the associated recruitment challenges; therefore, this study should be regarded as a preliminary investigation and the exploratory nature of the results should be underscored. Taken together, previous research indicates that GDF15 may have potential as a biomarker related to cognitive decline and neurodegenerative processes in several neurological conditions; however, its relevance in CD remains uncertain and warrants further investigation.

## 5 Conclusions

Despite the elevated GDF15 concentrations observed in individuals diagnosed with CD in comparison to healthy controls, no consistent correlations with cognitive performance were demonstrated, with the exception of a correlation with the visuospatial MoCA sub score. Furthermore, no associations were identified with respect to clinical characteristics or treatment response. It is important to emphasize that GDF15 cannot yet be regarded as a reliable biomarker of cognitive impairment in CD, as these findings are exploratory and require larger, methodologically robust studies to further elucidate its potential relevance in this rare disease.

## Data Availability

The original contributions presented in the study are included in the article/supplementary material, further inquiries can be directed to the corresponding author/s.
